# Individual Tree Segmentation Method Based on Mobile Backpack LiDAR Point Clouds

**DOI:** 10.3390/s21186007

**Published:** 2021-09-08

**Authors:** Lino Comesaña-Cebral, Joaquín Martínez-Sánchez, Henrique Lorenzo, Pedro Arias

**Affiliations:** Applied Geotechnology Group, CINTECX, Universidade de Vigo, 36310 Vigo, Spain; linojose.comesana@uvigo.es (L.C.-C.); hlorenzo@uvigo.es (H.L.); parias@uvigo.es (P.A.)

**Keywords:** TLS, individual tree, segmentation, DBSCAN, clustering, forest inventory

## Abstract

Individual tree (IT) segmentation is crucial for forest management, supporting forest inventory, biomass monitoring or tree competition analysis. Light detection and ranging (LiDAR) is a prominent technology in this context, outperforming competing technologies. Aerial laser scanning (ALS) is frequently used for forest documentation, showing good point densities at the tree-top surface. Even though under-canopy data collection is possible with multi-echo ALS, the number of points for regions near the ground in leafy forests drops drastically, and, as a result, terrestrial laser scanners (TLS) may be required to obtain reliable information about tree trunks or under-growth features. In this work, an IT extraction method for terrestrial backpack LiDAR data is presented. The method is based on DBSCAN clustering and cylinder voxelization of the volume, showing a high detection rate (∼90%) for tree locations obtained from point clouds, and low commission and submission errors (accuracy over 93%). The method includes a sensibility assessment to calculate the optimal input parameters and adapt the workflow to real-world data. This approach shows that forest management can benefit from IT segmentation, using a handheld TLS to improve data collection productivity.

## 1. Introduction

Forests are considered one of the biomes on the Earth’s land surface and include the most heterogeneous ecosystems around the globe. The area covered by primary forests has dramatically decreased since 1990 by more than 80 million hectares, whereas other areas of more than 100 million hectares are affected by issues such as wildfires, pests, invasive species and adverse extreme events that result in a decline in biodiversity [1]. Biodiversity conservation needs forest management based on guidelines that include the maintenance and monitoring of ecosystem connectivity, landscape heterogeneity and forest stand structural complexity [2]. Key elements for forest management include preserving protected and production areas, taking into consideration the spatial and temporal handling of harvest units, fire management and how these procedures affect forest structure and species composition [2,3]. In addition, silvicultural systems under climatic change have consequences on production, biodiversity and protection against natural hazards, resulting in a need for improved alternative scenarios for management [2,4].

In this context, improved forest management needs spatial and temporal monitoring systems, using remote sensing data to gather timely information and reduce costs [5]. Individual tree detection (ITC) is a key process that enables the automatic detection of changes in critical indicators and supports the actions to minimize their potential impact [6].

One of the most common technologies used for this purpose is laser scanning (LS), which is an active remote sensing technology that provides distance measurements between the observer (such as an aircraft or ground-based) and the surface illuminated by the laser beam [7,8]. The multiple benefits obtained with these systems have led to the rapid application of airborne laser scanning (ALS) to forest inventory [9].

However, laser scanning is still insufficiently applied for some purposes, due to the inadequate individual tree segmentation capability offered by LiDAR technology, caused by the complex patterns of forest canopies (giving significant omission and commission errors frequently) [10]. A segmentation process can be understood as a process for identifying and grouping objects based on statistical similarities or similar features in data [11]. Segmentation in 2D remote sensing data has been used since long ago [12], and there are many methods based on pixel, edge, region and graph identification for image analysis [13,14].

In addition, there are individual tree segmentation methods based on canopy estimation that were implemented for 3D LiDAR data obtained by ALS [15,16,17]. The application of these methods to point clouds collected by terrestrial laser scanners (TLS) often fails due to the lack of good point densities at the top-canopy surface. The occlusion effect still limits the processing efficiency to extract forest attributes [18], making it difficult to identify the tree top and other useful features to obtain a proper segmentation result [19]. Consequently, the classification method strongly depends on the data acquisition source [20].

Clustering methods in tree segmentation of TLS data have been widely used [21,22,23,24]. However, the method presented here shows a new individual tree extraction procedure based on an iterative DBSCAN clustering analysis of cylinder voxelizations that can be applied in any forest context. The basics of this algorithm relies on the trunk identification; therefore, the wider and more separated the trunks are in the point cloud acquired, the better the quality of the segmentations will be. Nevertheless, in the last section, a sensibility study of the different input parameters will be made in order to exploit the potential of the method presented here.

The objective of this work consists of a IT segmentation method based on TLS data collected by using a backpack mobile system. This paper is organized as follows: the next section illustrates the instrumental equipment used, the study area and the methodology of the segmentation algorithm, and Section 3 reports the experimental results and a discussion of their significance. The last section includes the main conclusions of the work along with future work.

## 2. Materials and Methods

### 2.1. Instruments

**Figure 1:**
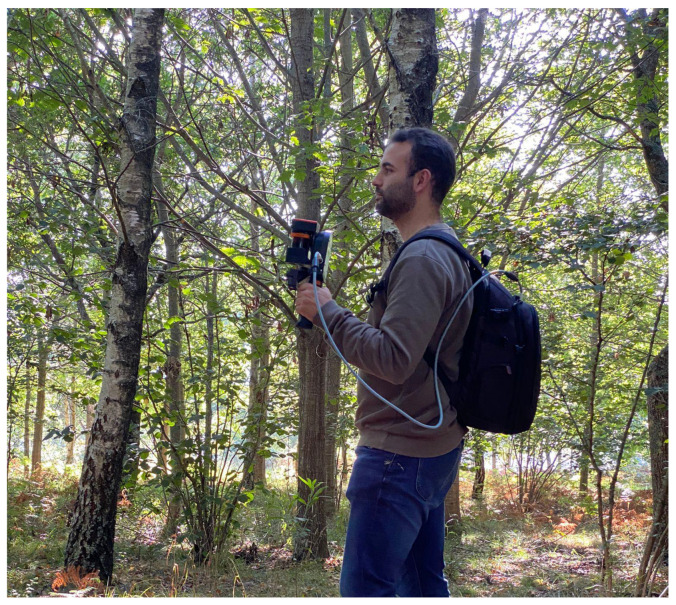
Backpack LiDAR system used during the data acquisition in the study area.

In this study, a ZEB-GO handheld laser scanner (view Figure 1) is used for the data acquisition. The technical specifications of this system are shown in Table 1.

The ZEB-GO handheld system consists of a 2D laser scanner (class 1 laser of 905 nm wavelength), combined with an inertial measurement unit (IMU). These are both mounted on top of a spring, itself mounted on a hand grip [25]. The laser specifications cite a ≤30 m measurement range, but this is unlikely to be achieved outdoors (due to ambient solar radiation) [18]; a survey swath of up to 15–20 m around the instrument is more realistic [18,26]. The hand-held part of the scanner (0.85 kg) is linked to a data logger carried in a backpack. As the user carries the ZEB-GO and walks through the environment, the scanner head swings back and forth, creating a 3D scanning field with data being captured at the speed of movement. The scanner is a time-of-flight (TOF) laser with a rate of 43,000 points/s and a field of view of 270${}^{\circ }$ horizontally and approximately 120${}^{\circ }$ vertically. No additional information about the intensity of the returned signal is collected.

This system includes a backpack system that can automatically collect and register data that are hard to access via standard GPS technologies, such as in underground cavities [27,28] or indoor buildings [29]. Due to the fact that under-canopy forests are considered GPS-denied areas, the TLS obtains its position and orientation based on IMU information and the LiDAR data collected by a Hokuyo system. A simultaneous localization and mapping (SLAM) approach supports the accurate mapping of the forest. The concept of SLAM is that a robot can be placed in an unknown environment and has the ability to create a map and then navigate to a particular destination [30]. The fact that the ZEB-GO is lightweight (850 g) and has no reliance on GPS makes it an ideal data capture method for inaccessible areas, such as under tree canopies and indoors [30,31], as shown in the right panel of Figure 1.

The motion created when the operator walked through the forest is an important part of the measurement technique [32], so a walking survey path between all possible trees in the region of study was followed in order to minimize occluded areas, as done in [18,32,33], based on the results of [33].

### 2.2. Study Area

**Figure 2:**
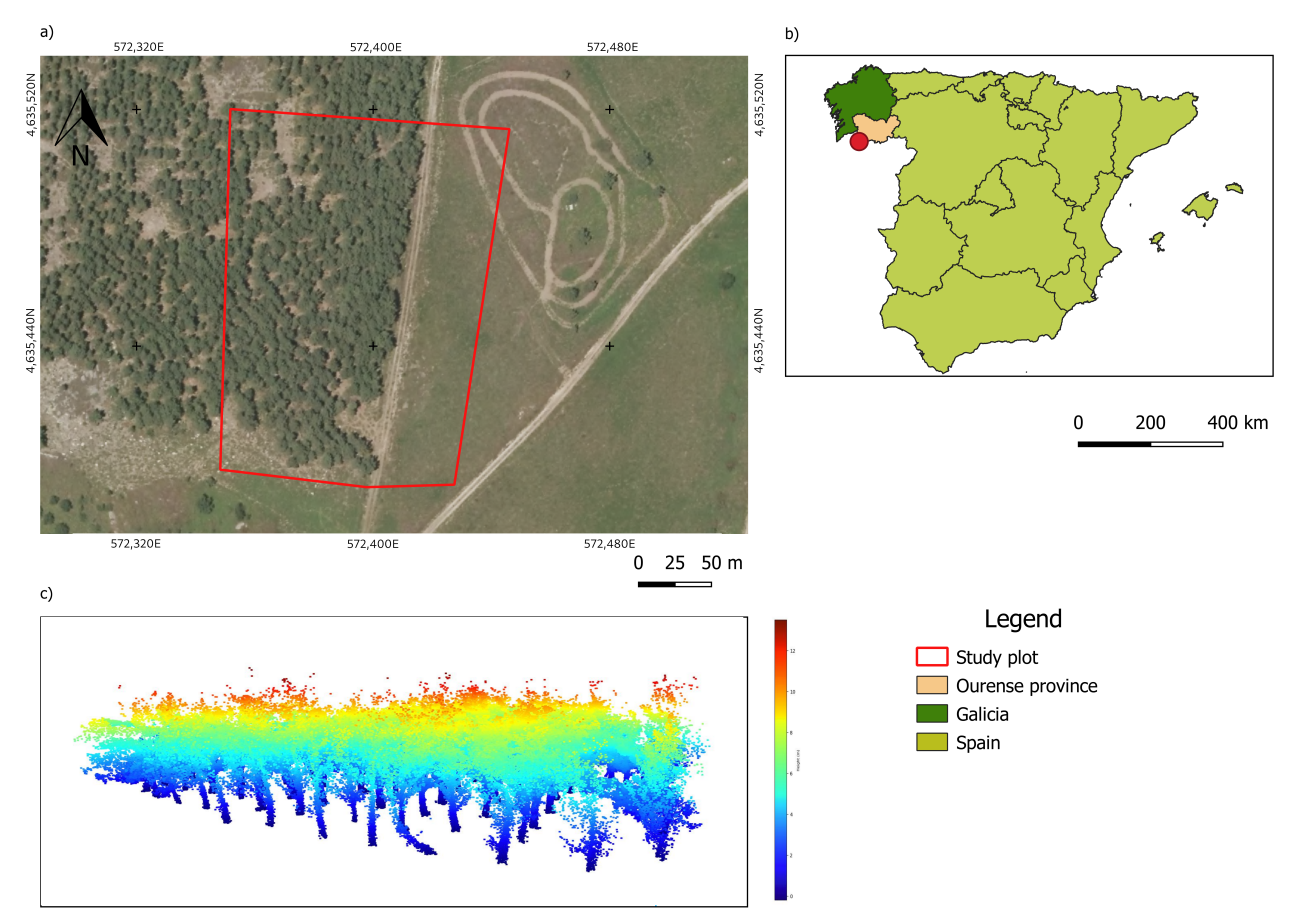
(**a**) Location of the plot in the study area; (**b**) location of the study area in the province of Ourense (Spain); (**c**) collected point cloud.

The equipment was used in the region of O Xurés, Galicia, Spain (−8.1599714; 41.9075281) as shown in Figure 2. It belongs to the Natural Park of Baixa Limia-Serra do Xurés, which was cataloged as an area for special conservation (ASC) [35]. The flora of the park are characterized by a deciduous forest, where the main tree species are *Quercus pyrenaica, Betula alba, Quercus suber, Arbustus unedo, Sorbus aucuparia* and *Ilex aquifolium*, which share the space with several endemic plants, including *Portuguese laurel* and *Prunus lusitanica* [35].

### 2.3. Methodology

**Figure 3:**
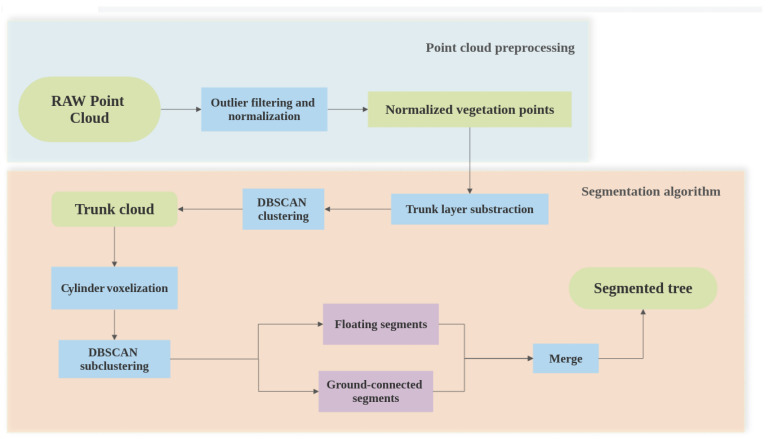
Workflow for the IT segmentation.

After the surveying is conducted, a raw point cloud of the plot is obtained from the TLS. In order to obtain the IT location from this raw point cloud, the methodology consists of a sequence of four steps, as follows: (i) outlier filter based on the trajectory of the handheld TLS, (ii) trunk layer subtraction, (iii) cylinder voxelization and clustering, and (iv) merging of floating segments and noise filtering. This workflow is schematically shown in Figure 3.

#### 2.3.1. Outlier Filter

The main purpose of the outlier filter is to erase those points in the raw point cloud that lie out of the plot to be considered. This points belong to branches, trunks or shrubs that are considered noise.

**Figure 4:**
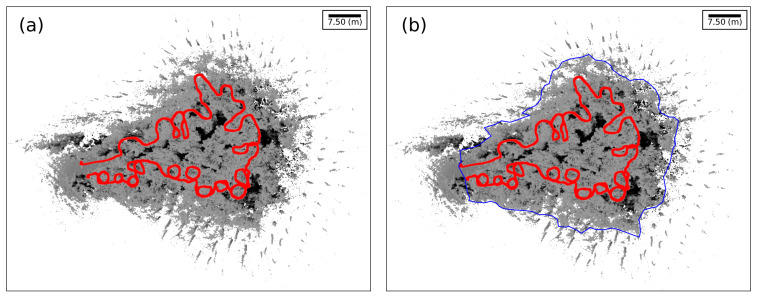
(**a**) Full original point cloud with trajectory marked in red color. (**b**) Polygon reconstruction from a trajectory’s convex hull in blue color.

Since the outliers are collected near the limit of the LS range (in our case, ∼30 m of diameter), a direct gate filter is applied to the raw point cloud, taking into account the trajectory of the TLS as a reference. A polygon is derived from the convex hull of the trajectory, filtering all points outside the polygon beyond a distance threshold (Figure 4).

After this transformation, the resulting point cloud is normalized with respect to the digital terrain model (DTM). Point cloud normalization is applied following the procedure presented in [36].

A morphological filter is applied to separate ground points from non-ground points using dilation and erosion operations to the height profile [37]. The resulting ground points are interpolated by inverse distance weighting (IDW) and meshed to obtain the DTM layer. Finally, using the DTM layer, the point heights are normalized.

**Figure 5:**
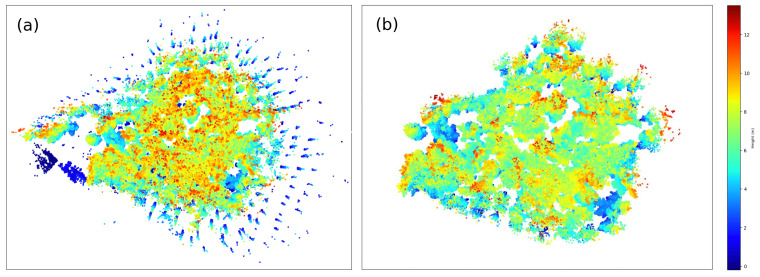
(**a**) Raw point cloud. (**b**) Outlier removal of the original point cloud.

Figure 5 shows the original raw point cloud and the normalized point cloud after outlier filtering and height normalization.

#### 2.3.2. Trunk Layer Subtraction

**Figure 6:**
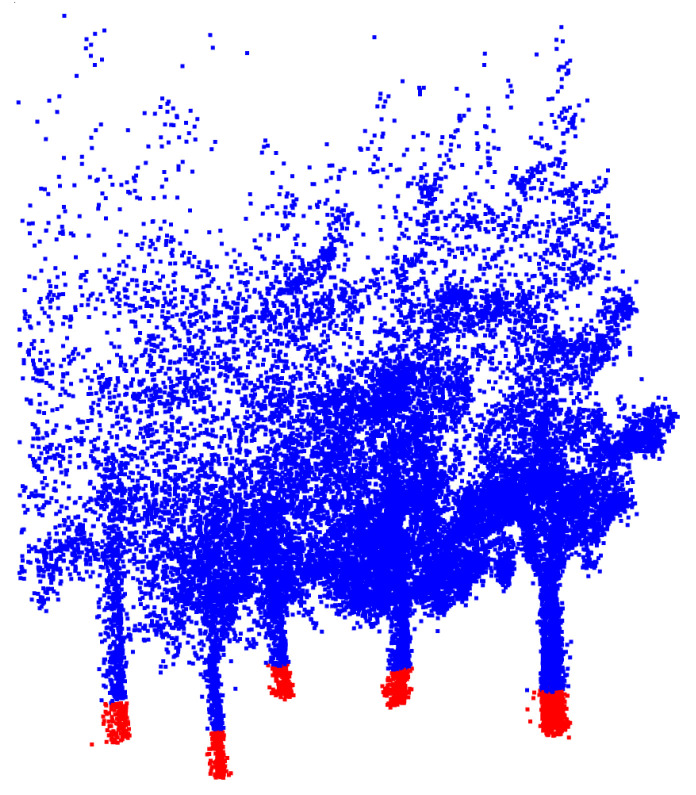
Partial screenshot of the point cloud with trunk cluster candidates colored in red.

After normalization, the point cloud includes vegetation points, such as shrubs, tree trunks and tree crowns. The first step for an individual tree detection in this method consists of creating a virtual horizontal layer at a certain height that is labeled as the *trunk layer*. After this layer is defined, we label those points belonging to the layer as *trunk points*, as shown in Figure 6. Depending on the height of the *trunk layer*, there might be a shrub or low crown points mislabeled as *trunk points*.

**Figure 7:**
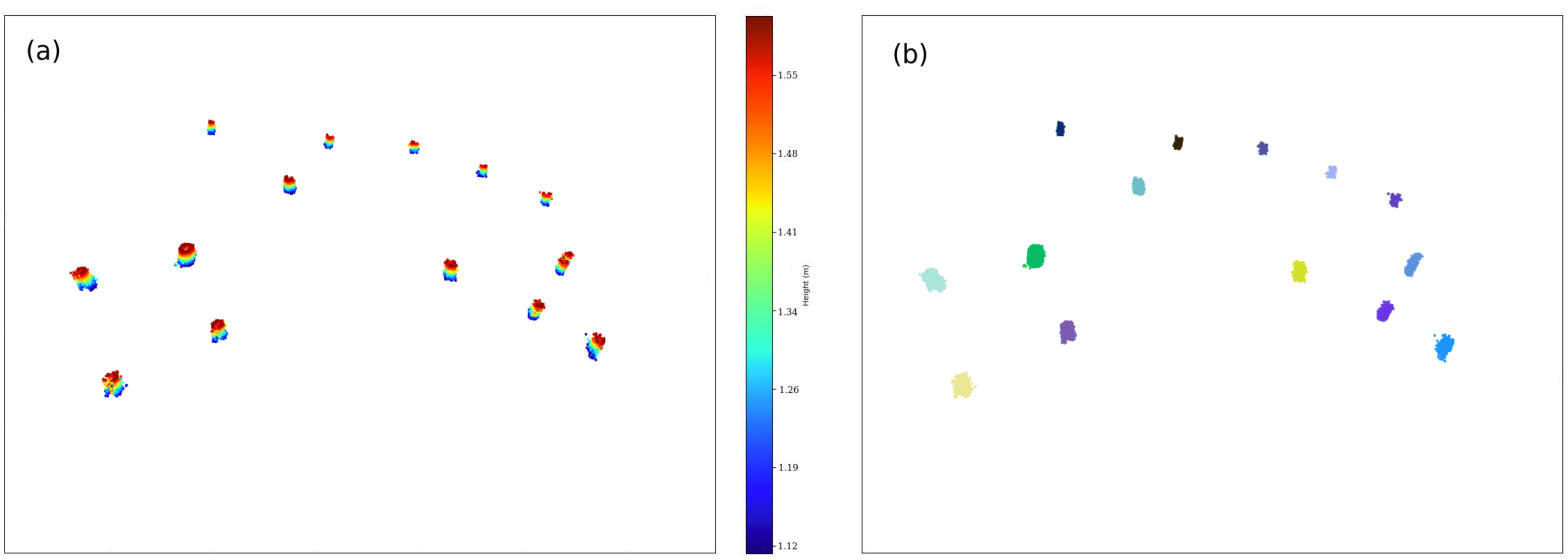
(**a**) Trunk layer subtraction. (**b**) Individual trunk identification via DBSCAN.

In order to filter these points as trunk candidates, a clustering analysis of the *trunk points* is carried out based on the DBSCAN algorithm [17]. The corresponding result for the trunk layer clustering analysis is shown in Figure 7.

DBSCAN clustering analysis is defined by three input parameters: the whole point cloud set, a point-neighboring radius ϵ and the minimal points per cluster, $N_{min}$. In this step of the algorithm, we define $\epsilon_{trunk}$ as the DBSCAN radius for the trunk cluster identification. As described in the following section, a sensibility assessment of the parameters is carried out to obtain the optimal parameters for the DBSCAN clustering analysis of the point cloud.

**Figure 8:**
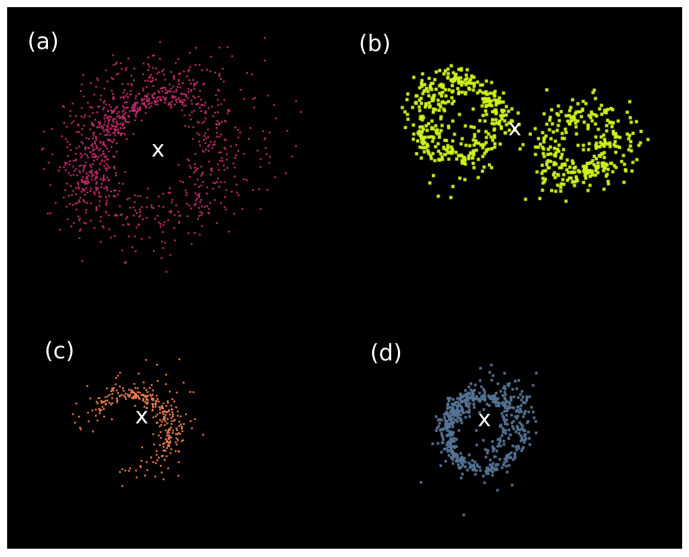
(**a**–**d**) Cross sections of some trunk clusters with its gravity centers marked as white crosses.

After clustering, we finally calculate the centroid for each *i* trunk in the XY plane, thus obtaining an estimation for the tree locations. For this, as shown in Figure 8, we set the same statistical weight for all *n* points in the trunk cluster and compute its center of mass as follows:

(1) $${\vec{c}}_{i}=\frac{1}{M}\sum_{k=1}^{n}m_{k}\cdot {\vec{r}}_{k},$$

where ${\vec{r}}_{k}\equiv {\vec{r}}_{k}\left(x,y\right)$ stands for the coordinates in the XY plane of the *k*-point and

(2) $$M=\sum_{k=1}^{n}m_{k}$$

is the total mass of the cluster. Thus, we have a continuous mass distribution, the center of mass being the same as the geometrical center of the total volume [38].

However, in the case of two close trunks, if the $\epsilon_{trunk}$ used is big enough, both trunks are considered as only one, leading to a wrong tree identification. This example can be seen in Figure 8b, where two trunks are close and the DBSCAN clustering merged them in one cluster. This phenomenon is mainly responsible for the omission errors ($E_{OM}$) during the algorithm development; a discussion about this fact is made in the last sections.

Additionally, points of the ZEB-GO slice appear to be within and outside the stem, and the density of points around the trunk follows a Gaussian shape with a model located in the outline of the cross section [18] (Figure 8). This is an important factor to take into account for making a proper noise filtering.

#### 2.3.3. Cylinder Voxelization

Cylinder voxelization supports the segmentation of the point cloud in clusters belonging to the same tree.

Once the trunk centroid estimations (i.e., the tree locations) are obtained, the closest neighbor of each trunk is derived. The distance between a trunk and its closest neighbor is defined as the *candidate radius* of the cylinder to be analyzed for segmentation.

**Figure 9:**
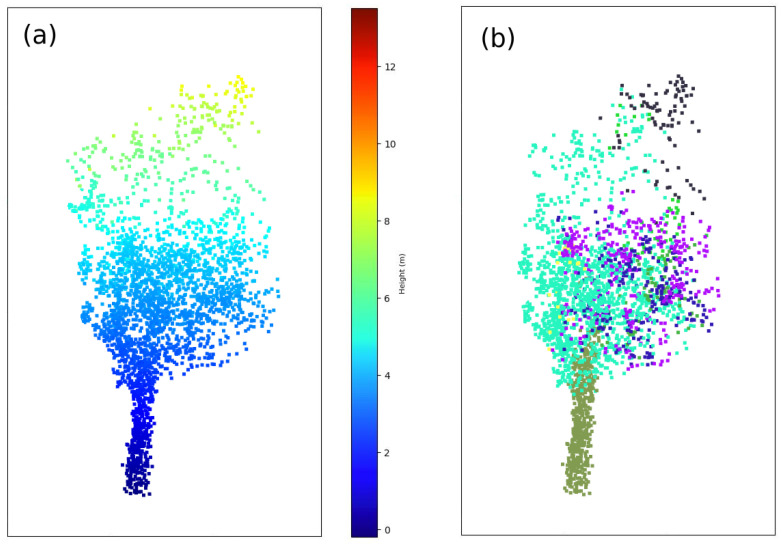
(**a**) Cylinder cloud subtracted. (**b**) Subclustering of the cylinder point cloud.

Considering the trunk centroid as the center, an infinite cylinder with the *candidate radius* is calculated. All the points inside this cylinder are accordingly labeled as *cylinder points* and segmented as $C_{i}$ point cloud. For each cylinder point cloud $C_{i}$, a new DBSCAN clustering analysis is performed. In this DBSCAN procedure, analogous to the trunk layer subtraction step, we define the neighbor DBSCAN radius as the $\epsilon_{cylinder}$, and low values for it are expected in order to reject the points of the surrounding tree crowns (it is preferably to have some small-sized clusters instead of only one big cluster because the next step consists of merging all of the subclusters, following the constraint that not all subclusters in a cylinder voxelization belong to the same tree). As a result, it is likely to obtain an oversegmentation with *j* subclusters $C_{i}^{j}$ inside the candidate *cylinder-points* in $C_{i}$ instead of only one subcluster. An example of a cylinder point cloud and its DBSCAN subclustering can be seen in Figure 9, where a small value of $\epsilon_{cylinder}$ is set in order to have many subclusters in one cylinder voxelization.

This is an iterative process, where all the candidate *cylinder-points* are filtered and not considered in the next iteration, in order to save the computational resources. The process is stopped when there are no pending trunks to label. Points that do not belong to any of the cylinders are labeled as *discarded points* in the cylinder voxelization.

#### 2.3.4. Merging the Floating Segments and Noise Filtering

After cylinder voxelization and subclustering, two types of clusters are expected: ground-connected and floating segments as shown in the right panel of Figure 9. In order to label the subclusters, the vertical continuity is assessed by comparing their point density at different heights. In the case that a discontinuity is detected between the ground and the *candidate subcluster* $C_{i}^{j}$, the *candidate subcluster* is labeled as a floating segment. Otherwise, the *candidate subcluster* is labeled as being ground-connected.

For the aerial LiDAR data acquired, this would lead to an incorrect performance, due to the low point density under the tree crowns, showing trees with few trunk points. However, in our case, as the algorithm is developed for terrestrial LiDAR, this issue is completely avoided, and for a real tree, there will be always at least one segment very close to the ground that is considered ground-connected.

**Figure 10:**
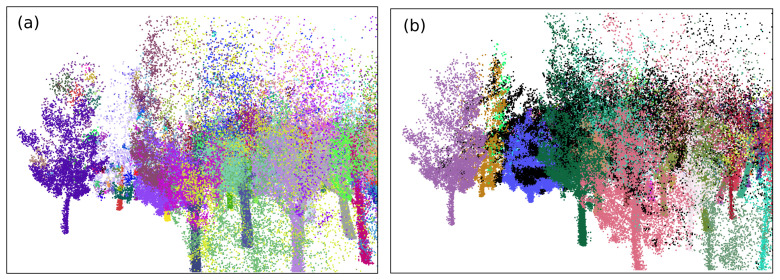
Screenshots of the point cloud at this stage. (**a**) The whole set of points are classified into floating and ground-connected clusters. (**b**) All segments are merged, following the logic explained in this work (black points represent all the points that are discarded during the cylinder voxelization and will be correctly assigned to each segment in the last step).

Once all subclusters are classified, merging the floating segments consists of assigning them to the closest ground-connected segment. In Figure 10, there is an example of the point cloud’s state before and after this process. In the left panel, there are some groups of points that do not seem to match their correspondent tree, i.e., the floating segments, so after identifying them by evaluating their vertical continuity, they are merged with the closest ground-connected segment. After repeating this process with all cylinder point clouds, there will be a remaining group of points without any classification yet. We will call them *discarded points*, and their analysis will be the last step of the algorithm’s procedure.

To finish, the *discarded points* in the cylinder voxelization are studied. In the trunk layer subtraction step, all trunks are identified by DBSCAN clustering providing the location of all trees and, then, for each *i* trunk (with gravity center $c_{i}\left(x,y\right)$) the closest *j* trunk cluster (with gravity center $c_{j}\left(x,y\right)$) within a threshold is searched. Once the *j* trunk is found, a cylinder of radius $d_{ij}=\left|c_{i}-c_{j}\right|$ centered in $c_{i}\left(x,y\right)$ is made and all points of the whole point cloud set, *C*, that lies in that cylinder are stored, creating the cylinder voxelization $C_{i}$ set.

**Figure 11:**
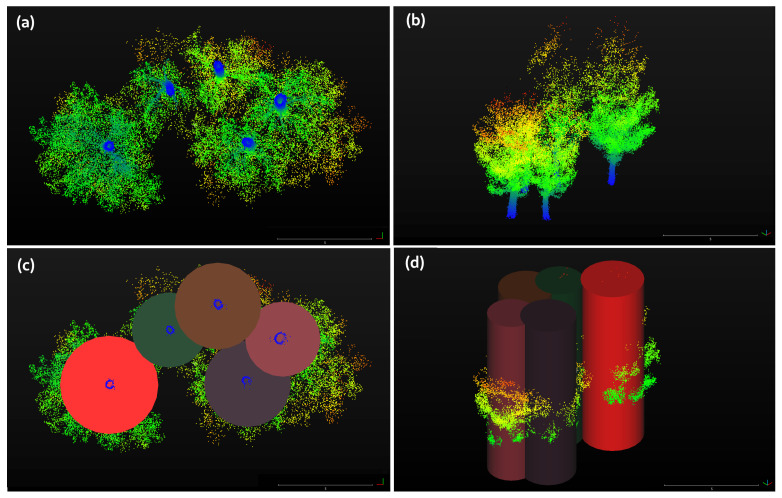
(**a**,**b**) Two point of views of some manually subtracted trees for the point cloud. (**c**,**d**) Cylinder envelope of all points inside the voxelization. All points that are not inside any cylinder and, thus, discarded in the voxelization, are analyzed in the last step of the workflow.

Iterating over all *i* trunk locations, we have that the labeled points consist of the following:

(3) $$C\geq \overset{N}{\underset{i=1}{⋃}}C_{i},$$

with *N* being the number of total segments identified. However, the whole point cloud set *C* can be greater than the union of all $C_{i}$ individual tree sets because some points are discarded during the voxelization step. As shown in Figure 11, the red cylinders union overlaps only points close by their original tree, even if some points were not associated yet and, thus, did not belong to any cylinder voxelization. If we define *discarded points* as $C^{*}$, one can show the following:

(4) $$C^{*}\equiv C-\left(\overset{N}{\underset{i=1}{⋃}}C_{i}\right)$$

and, thus, the *discarded points* are those outside the union of all cylinders. These points are filtered and, if their distance to the closest segment set $C_{i}$ is below a threshold distance $d^{*}$, they are added to that segment and discarded otherwise.

At the end of this process, depending of the $d^{*}$ selected, all points that are not added to any segment set $C_{i}$ are associated to a noise set *N*. If a small threshold noise distance $d^{*}$ is used, then the noise set *N* is greater than in the case of selecting a bigger $d^{*}$. This parameter $d^{*}$ varies for each forest because it is related to the proximity of the trees, so establishing an optimal value for it is different for every datum acquired. In Section 3.2, a sensibility assessment is made and shows how to improve the goodness of the segmentation algorithm behavior.

### 2.4. Validation

The validation consists of defining and applying the metrics to obtain the performance of the method. The metrics found in the literature for ALS processing [10,39,40,41,42] are not applicable to our methodology because of the different point of view of the TLS. Specifically, the tree top or the maximum canopy width are not parameters of interest in our case. In order to measure the performance of the segmentation, we consider the segments as true positives (TP), false negatives (FN) and false positives (FP) based on the following iterative process.

First, we subtract all trees in the processed point cloud by hand and, for each manually segmented tree, a centroid of its trunk in the XY plane is computed following the same logic as in Section 2.3.2. Those centroids are considered to be each true tree location. Once all real trees are identified, the iterative validation process starts.

For each manually subtracted tree, the closest tree is searched and the distance between them is defined as $d_{min}$. This distance is calculated as the average of all the distances between the corresponding points, obtaining an estimation of the clusters proximity. Then, we search the closest segmented tree from the algorithm (from now, it will be referred to as an algorithm tree) within a distance $d_{min}$. If any algorithm tree is not within that radius, then that real tree is a false negative (FN) and is not taken into account in further iterations.

In the case that there is at least one algorithm tree within a radius $d_{min}$, the numbers of points that it has will be outlined as $N^{*}$. In addition, the number of points of the iterated real tree and its closest manually subtracted are defined as $N_{1}$ and $N_{2}$, respectively.

Then, the next comparison process starts:

- If $N^{*}>N_{1}$ and $N^{*}\geq N_{1}+N_{2}$:
  The algorithm tree overlaps 2 real trees (or more), so it is considered a false positive (FP) and will not be taken into account anymore.
- If $N^{*}>N_{1}$ and $N^{*}<N_{1}+N_{2}$:
  The algorithm tree is in coincidence with the real tree and contains some more points than it should, so it is considered a true positive (TP) and will not be taken into account anymore.
- If $N^{*}\leq N_{1}$:
  The algorithm tree is in coincidence with the real tree but may be incomplete comparing both of them. In other words, the majority of the algorithm tree points is truly identified, but the real tree still has more that would be associated with the other algorithm tree, so it is considered a true positive (TP) and will not be taken into account anymore.

**Figure 12:**
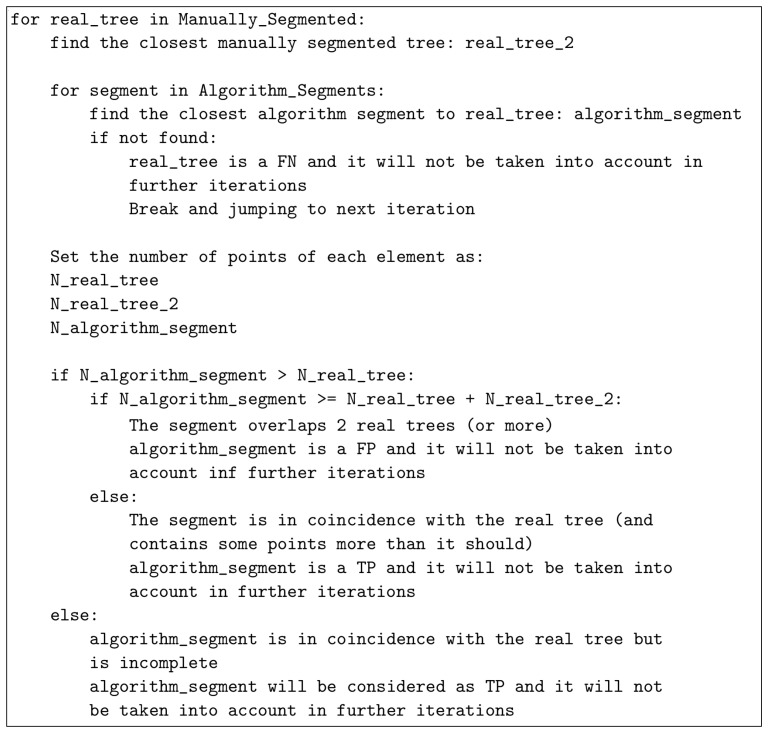
Iterative process used during the validation.

Schematically, the validation process follows the scheme shown in Figure 12. Applying this metric, we define the following confidence estimators [40]:

(5) $$DR=\frac{N_{TP}}{N_{R}},$$

(6) $$E_{COM}=\frac{N_{FN}}{N_{TP}+N_{FN}},$$

(7) $$E_{OM}=\frac{N_{FP}}{N_{TP}+N_{FP}},$$

where DR, $E_{COM}$ and $E_{OM}$ are the detection rate, the commission error and the omission error, respectively. In addition, $N_{TP}$ is the number of trees truly detected (TP), $N_{R}$ is the number of real manually subtracted trees, $N_{FN}$ is the number of trees classified as false negatives (FN) and $N_{FP}$ is the number of trees classified as false positives (FP).

Omission errors occur when a real tree is not truly detected when it should be. On the other hand, commission errors are the opposite; they appear when an algorithm segment is classified as a real tree when it should not be. As mentioned in Section 2.3.2, making a good identification of each tree trunk and, then, of each real tree minimizes the omission error. The floating segments merging step is a good procedure for minimizing the commission error because most of the floating segments that could be identified as a real tree are combined with the closest ground-connected tree (that would be a real tree because it has a trunk), and this fact leads to a small commission error.

Summarizing, DBSCAN parameters, such as $\epsilon_{trunk}$ and $\epsilon_{cylinder}$ and the threshold distance $d^{*}$ for noise filtering, are the most important factors because the performance of the algorithm strongly depends on them, and establishing reliable references of this factors will help to minimize omission and commission errors and maximize the detection rate, as will be shown in the sensibility assessment of the next section.

## 3. Results and Discussions

### 3.1. Segmentation of Backpack Point Clouds

**Figure 13:**
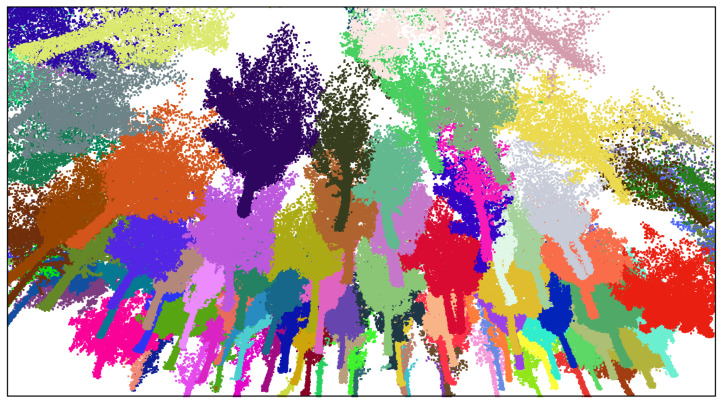
Zoom screenshot at the segmented point cloud.

**Figure 14:**
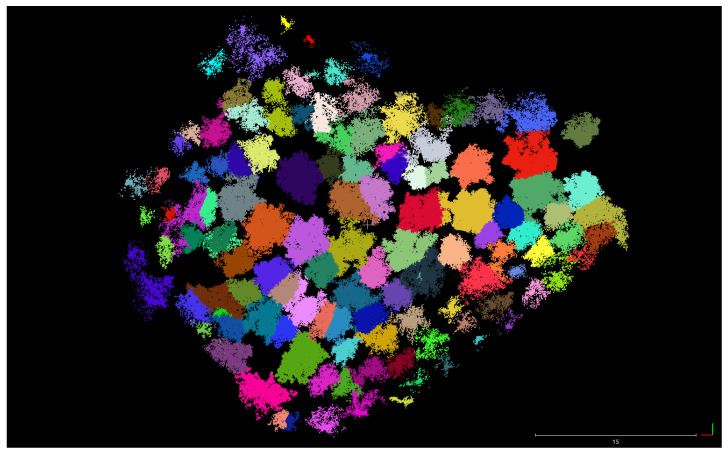
Orthographic view of the whole point cloud segmented (geometrical scale in meters).

**Figure 15:**
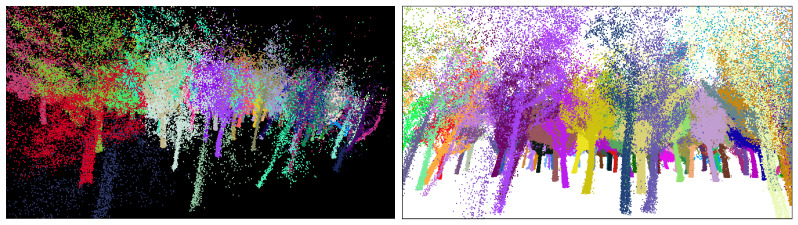
Zoom screenshots at the segmented point cloud.

As previously mentioned, the region of study was in O Xurés (Galicia, Spain), and the point clouds consist of ∼3 million points. We implemented the methodology in a Python script [43] with Open3D library for 3D data processing [44] to extract IT with both qualitative and quantitative good results. Processing times of the program were timely consistent for the point cloud taken (in the range of 3–8 min), in comparison with other similar methods such as those in [24,45,46,47], where times were in the order of 2 days, 6.22 min, 4 min and 4.78 min, respectively. In addition, the times of processing in this work can be improved by varying the input parameters given. Some examples are shown in Figure 13, Figure 14 and Figure 15.

### 3.2. Validation and Sensibility Assessment

**Figure 16:**
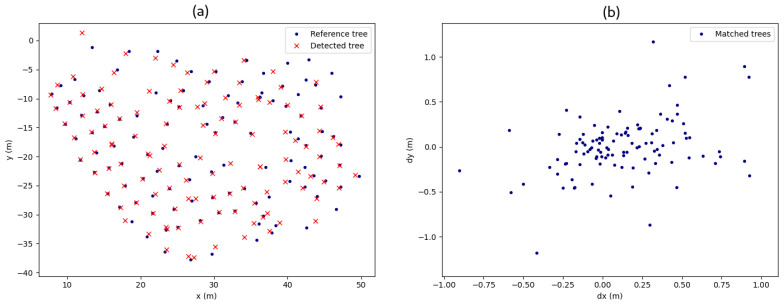
(**a**) A comparison between each subtracted tree and its reference tree. (**b**) Positional accuracy plots.

To validate this algorithm, we select one of the point clouds of O Xurés and segmented it by hand. This is important to test how many trees were effectively identified. Once the point cloud is manually segmented, each tree position is located by iterating over all manual segments and calculating the centroid of the points at the beginning of each trunk. The location of the segmented trees is evaluated, following the metric explained in the methodology, labeling each tree as TP, FP or FN. When a segment is considered a TP, we mark it as a matched tree, as shown in the left panel of Figure 16. In addition, a scattering plot of the deviation of each segment from its actual tree is shown in the right panel of Figure 16, showing low errors in the identification process. There are also some matched trees with location errors over 1 m, but this is because some segments are considered TP when they overlap parts of their closest trees, creating the trunk centroid far from its real location. The tree is regarded as a TP because it is effectively detected, but the subtracted tree location contributes to make scatter errors in plot (b) of Figure 16.

Equations (3)–(5) help to achieve the best input parameters for the segmentation algorithm. These input parameters are those used during the algorithm’s development: the DBSCAN radius of the trunk identification ($\epsilon_{trunk}$), the DBSCAN radius used inside each cylinder voxelization ($\epsilon_{cylinder}$) and the threshold noise distance that filters all *discarded* points during the cylinder voxelization step ($d^{*}$).

**Figure 17:**
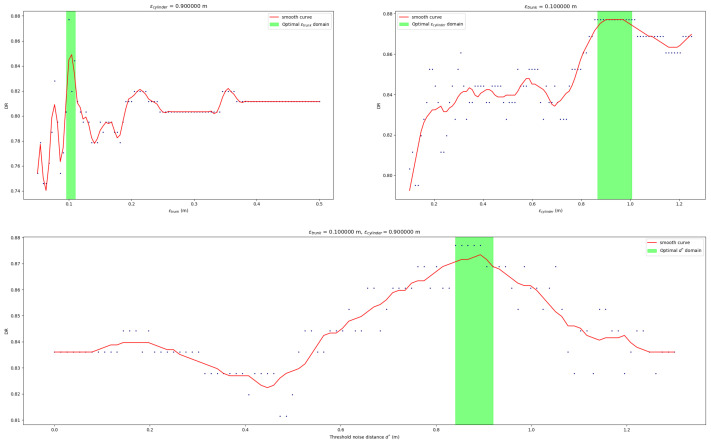
Sensibility analysis of the different input parameters.

As previously mentioned, DBSCAN constraints and threshold noise distances depend on the case study, but the proposed estimators, resulting from the sensibility analysis, support the setup of the optimal parameters as shown in Figure 17.

In the study area, $\epsilon_{trunk}$ (upper left panel in Figure 17) shows more sensibility to little variations than other input parameters. We set the optimal value for $\epsilon_{trunk}=0.1$ m because it is the best detection rate for that scenario. However, we set the maximum limit for $\epsilon_{trunk}$ as 0.5 m because a greater value would not be realistic for trunk-clustering analysis in our study area.

In other cases, for $\epsilon_{trunk}=0.1$ m, we could identify optimal windows for $\epsilon_{cilinder}=\left[0.8,1.0\right]$ m and a threshold noise distance of 0.87 m.

The best detection rate obtained was 86.885% ($E_{COM}=13.114\%$ and $E_{OM}=10.924\%$) for input parameters of $\epsilon_{trunks}=0.1$ m, $\epsilon_{cylinder}=0.9$ m and threshold noise distance of 0.87 m.

In general, algorithms for forest segmentation in UAV LiDAR point clouds have similar detection rates between them, varying from 26% to 96% (depending on the method and the type of data acquired) [15]. Individual tree detection from photogrammetric point clouds created from UAV data were tested, for example, by Sperclich et al. [48] who achieved DR near 90%. Other similar methods were applied like in [49], where the overall detection rate was 76.1% for a distance-adaptive search method of stem radius or in [22], where a single-scan TLS was applied in order to detect stems in a dense and homogeneous forest with a detection rate of 88%.

The main inconvenience detected in our method is the sensibility to $\epsilon_{trunk}$, as seen in the upper left panel in Figure 17. When two or more trunks are close enough, the cylinder voxelization assumes all trunks that fall inside the cylinder to be a unique tree. This situation may occur if the region of study has trees with very close trunks (O∼ cm). However, this situation is challenging, even for a human practitioner.

**Figure 18:**
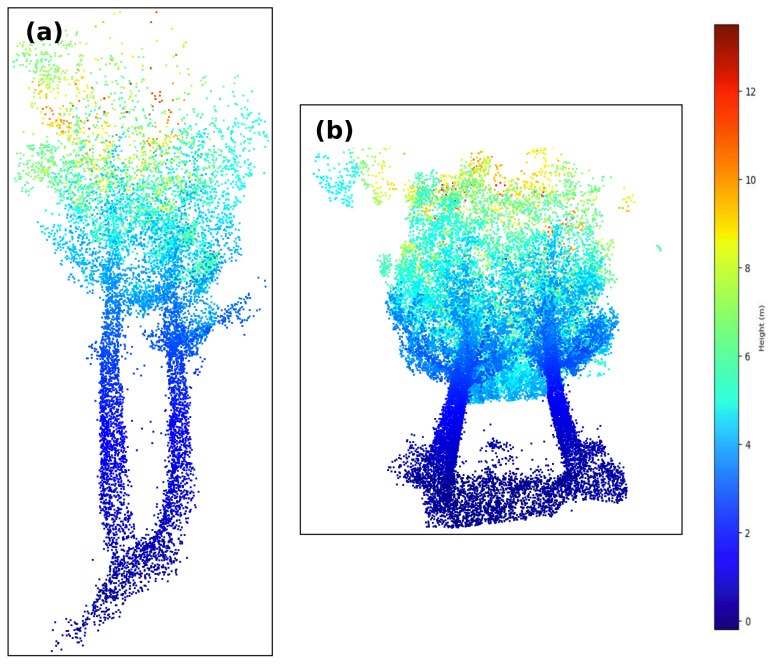
Examples of hard human classification trees. (**a**) Close trunks that can be interpreted as one tree or two. (**b**) Two trees with nearby canopies.

Another example is the canopy segmentation task: if there is more than one tree with nearby canopies, it will be difficult to identify which points belong to each tree. Both situations are shown in Figure 18.

## 4. Conclusions

Forest inventory from airborne laser scanning (ALS) has had a rapid application due to the feasibility of the path followed by an aerial vehicle. However, laser scanning can be a hard task for places where these instruments have difficulties in their flights, creating a lack of information in regions where the occlusion is big enough. In the literature, terrestrial laser scanning (TLS) is shown to be useful for accessing the under-canopy region but with low point density at the canopy surface.

Taking into account these facts from ALS and TLS data, some computational methods were developed in order to extract forest features, such as individual tree shapes or tree locations. Nevertheless, almost all methods are designed for specific types of measurement sources, making some algorithms work better with aerial or terrestrial LiDAR data.

The aim of this work consists of segmentation of individual trees (IT) from raw point clouds obtained by a backpack TLS in the Spanish region of O Xurés, Galicia, Spain. For this purpose, a segmentation algorithm was designed and successfully applied, leading high detection rates.

The method proposed needs a normalized point cloud as input data and provides the same cloud with all their points labeled with the IT they belong to. As shown in the previous section, the results obtained are good enough to show that this technology is useful for inventory tasks in forests, achieving detection rates near 90% and low commission and omission errors.

For the study area, the variation of the DBSCAN parameters and threshold distances was studied in order to minimize the omission and commission errors. One way to improve the detection rate is the implementation of a convolutionary neural network (CNN). Training this CNN with the individual tree segments of the backpack LiDAR data will lead to a more sophisticated and complex model that may reduce the computational calculus time. In addition, this network can help in the generation of a dataset by creating artificial trees point clouds by data augmentation.

Despite the obstacles mentioned in the validation assessment, the algorithm developed showed good results with undergrowth and completely wild nature environments, which is the basis for IT-level forest management. 

## Figures and Tables

**Table 1:** Technical specifications of the ZEB Go handheld laser scanner from [34].

Range	20 m (features < 15 m)
Laser	Class 1/λ 905 nm
FOV	360${}^{\circ }$ × 270${}^{\circ }$
Scanner weight	850 g
Scanner points per second	43,000
Number of sensors	1
Relative accuracy	1–3 cm (environment dependent)
